# Novel lncRNAs LINC01221, RP11-472G21.2 and CRNDE are markers of differential expression in pediatric patients with T cell acute lymphoblastic leukemia

**DOI:** 10.1186/s12935-024-03255-y

**Published:** 2024-02-09

**Authors:** Pankaj Sharma, Parminder kaur, Prateek Bhatia, Amita Trehan, Sreejesh Sreedharanunni, Minu Singh

**Affiliations:** 1 Hematology-Oncology Unit, Department of Pediatrics, Postgraduate Institute of Medical Education and Research, Chandigarh, India; 2 Department of Hematology, Postgraduate Institute of Medical Education and Research, Chandigarh, India

**Keywords:** T cell acute lymphoblastic leukemia, Non-coding RNA, Transcriptome, B-cell acute lymphoblastic leukemia

## Abstract

**Introduction:**

Pediatric T-cell acute lymphoblastic leukemia (T-ALL) poses significant challenges due to its aggressive nature and resistance to standard treatments. Long non-coding RNAs (lncRNAs) have emerged as potential biomarkers and therapeutic targets in leukemia. This study aims to characterize the lncRNA landscape in pediatric T-ALL, identify specific lncRNAs signatures, and assess their clinical relevance.

**Methods:**

RNA sequencing was performed on T-ALL patient and control samples. Differential expression analysis identified dysregulated lncRNAs and mRNAs. Functional enrichment analysis revealed potential roles of these lncRNAs in cancer pathogenesis. Validation of candidate lncRNAs was conducted using real-time PCR. Clinical correlations were assessed, including associations with patients’ clinical characteristics and survival outcomes.

**Results:**

Analysis identified 674 dysregulated lncRNAs in pediatric T-ALL, with LINC01221 and CRNDE showing the most interactions in cancer progression pathways. Functional enrichment indicated involvement in apoptosis, survival, proliferation, and metastasis. Top 10 lncRNAs based on adjusted p value < 0.05 and Fold Change > 2 were selected for validation. Seven lncRNAs LINC01221, PCAT18, LINC00977, RP11-620J15.3, RP11-472G21.2, CTD-2291D10.4, and CRNDE showed correlation with RNA sequencing data. RP11-472G21.2 and CTD-2291D10.4 were highly expressed in T-ALL patients, with RP11-620J15.3 correlating significantly with better overall survival (*p* = 0.0007) at a median follow up of 32 months. The identified lncRNAs were further analysed in B-ALL patients. Distinct lncRNAs signatures were noted, distinguishing T-ALL from B-ALL and healthy controls, with lineage-specific overexpression of LINC01221 (*p* < 0.0001), RP11-472G21.2 (*p* < 0.001) and CRNDE (*p* = 0.04) in T-ALL.

**Conclusion:**

This study provides insights into the lncRNA landscape of pediatric T-ALL, offering potential diagnostic and prognostic markers. RP11-620J15.3 emerges as a promising prognostic marker, and distinct lncRNAs signatures may aid in the differentiation of T-ALL subtypes. Further research with larger cohorts is warranted to validate these findings and advance personalized treatment strategies for pediatric T-ALL patients.

**Supplementary Information:**

The online version contains supplementary material available at 10.1186/s12935-024-03255-y.

## Introduction

Acute lymphoblastic leukemia (ALL) is the most common cancer in children and adolescents [1]. While 90% of children suffering from B acute lymphoblastic leukemia (B-ALL) become disease-free after five years, the prognosis is poorer for T-cell acute lymphoblastic leukemia (T-ALL) [2]. Despite the success of modern chemotherapy regimens and stem cell transplantation; 20–30% of patients relapse, and only 20% of relapsed T-ALL patients survive for five years [3].

The role of lncRNAs in cancer progression has been a focus of cancer research in the last decade [4]. LncRNAs are a type of RNA that are more than 200 bp in size and are not involved in protein-coding. lncRNAs primarily interact with mRNA, DNA, protein, and miRNA and consequently regulate gene expression at the epigenetic, transcriptional, post-transcriptional, translational, and post-translational levels in a variety of ways. They play important roles in biological processes such as chromatin remodeling, transcriptional activation, transcriptional interference, RNA processing, and mRNA translation [5–8].They have been described to play a role in normal biological functions as well as in various diseases including cancers [9]. LncRNAs are reported to be differentially expressed in various malignancies and play an important role in cancer progression, metastasis and chemoresistance [9]. In recent studies, lncRNAs signature has been involved in discriminating B- and T-Cell leukemia and in finding them as novel biomarkers and therapeutic targets in leukemia [10, 11].

Studies show that many lncRNAs that are dysregulated in leukemia play an important role in the development and progression of leukemia. Acute myeloid leukemia is the most studied acute leukemia with respect to lncRNAs [12]. CCDC26 is the first lncRNA reported in AML and has been shown to regulate leukemia cell proliferation by regulating *KIT* expression [13]. Another lncRNA involved in AML and shown to regulate expression of *KIT* is ZNF571-AS1 [14]. LINC00899 is reported to have the potential to be used as a biomarker for AML as its expression is frequently upregulated in AML patients [15]. LncRNA HOXB-AS3 and HOXBLINC are upregulated in NPM1mut AML patients and contribute to the development of leukemia blasts by regulating NPMI-EBP1 interaction [16, 17]. Further, studies have been reported the role of lncRNAs in B-ALL development and progression. Fernando and colleagues reported the initial evidence of overexpression of BALR-2 lncRNA in B-ALL patients. They demonstrated that BALR-2 increases leukemia cell proliferation and inhibits apoptosis through glucocorticoid regulation. Further, overexpression of BALR-2 is was also shown to correlate with poor overall survival and low response to prednisone treatment [18]. Among B-ALL, uc.112 also shows differential expression among patients with hyper diploidy as compared to other karyotype results [19]. In a study by Lopez B DA et al., lncRNAs LINC00152 and LINC01013 were also found to be differentially expressed in B-ALL patients with early relapse and early death. They showed that LINC00152 is able to mediate the cell-substrate adhesion and peptidyl–tyrosine auto phosphorylation related biological processes [20]. These studies suggest that various lncRNAs are differentially expressed in leukemia and can be used as prognostic markers or therapy targets. Though studies have reported a few lncRNAs to be upregulated in T-ALL such as ARIEL, LUNAR1, NALT etc., however such studies are limited due to low incidence of the disease [21–23].

Thus, in the present study, we have explored the lncRNAs expression profile in pediatric T-ALL patients and put forward the potential correlation of specific lncRNAs with disease outcome. We used RNA sequencing to characterize transcriptome-wide lncRNAs and mRNAs in T-ALL patients, and compared the data with control samples as well as T-ALL cell lines. In addition, we performed pathway enrichment and gene correlation studies using available databases and performed clinical correlation with patient characteristics. The goal of this study was to discover differentially expressed lncRNAs patterns and explore their potential involvement in childhood T-ALL.

## Methods

### Study Population

Newly diagnosed pediatric T-ALL and B-ALL cases (age ≤ 12 years) confirmed on morphology and immunophenotype (flow cytometry) were enrolled for the study. Cases were classified immunophenotypically by flow cytometry into immature (pro T- and pre T-), cortical and mature T-ALL based on the EGIL criteria [24]. ETP-ALL was recognized based on the previously defined criteria [25]. Patients were considered as good prednisolone responder at day 8 if absolute blast counts (ABC) were < 1000/ul and poor prednisolone responder if ABC > 1000/ul. Patients were treated and followed up uniformly as per the ICiCLe treatment protocol (Clinical Trials Registry-India number, CTRI/2015/12/006,434) [26]. Written informed consent in agreement with the Declaration of Helsinki was taken from children and/or guardians and the study was approved by the Institutional Ethics board.

### RNA sequencing

RNA was extracted from mononuclear cells isolated from patient’s blood samples by the RNA blood kit (Qiagen Inc.) as per the manufacturer’s protocol. NEBNext RNA Ultra II directional protocol was used to prepare the libraries for total RNA. Paired-end whole transcriptome sequencing was performed on the Illumina NovaSeq to generate 60 M, 2 × 150 bp reads/sample. The raw reads were filtered using Trimmomatic for quality scores and adapters. Filtered reads were aligned to Human genome (hg19) using splice aware aligner HISAT2 to quantify reads mapped to each transcript. Total number of uniquely mapped reads were counted using feature counts. LncRNAs annotations were done with lncBook. The uniquely mapped reads were then subjected to differential gene expression using Deseq2.

### Functional Enrichment Analysis

As the lncRNAs functions are poorly defined, we used the database lncSEA (version 2.0) [27] for the functional enrichment analysis of the lncRNAs and used NPinter (version 4.0) [28] and ENCORI [29] to predict the interaction of upregulated lncRNAs with other biomolecules. Enrichment analysis for correlated mRNAs were performed using EnrichR [30], Geneontology (GO) [31, 32] KEGG [33] and Reactome databases.

### Real time PCR

Total RNA was collected using Qiagen Blood RNA kit (Qiagen Inc.) and reverse-transcribed into cDNA using First Strand cDNA synthesis kit (Thermo Fisher Scientific). The primer sequences are given in supplementary table 1. Quantitative PCR analysis was performed on a QuantStudio 5 Real-Time PCR System (Applied Biosystems) using PowerUP SYBR Green PCR Master Mix (Thermo Fisher Scientific). The PCR primer sequences can be found in the Supplementary Information (supplementary table 1). Real Time PCR data analysis was performed by the Livak Method [34]. Median of fold change of each lncRNA was used for data analysis and clinical correlation.

### Outcome assessment and statistical analysis

Treatment outcome parameters analyzed included relapse free survival (RFS) or relapse rate (RR) - defined as time period from onset of therapy to disease relapse for those achieving complete remission with censoring at death in remission or last contact. Overall survival (OS)- defined as time period from onset of therapy to death with censoring at last contact. Event free survival (EFS)- defined as time period from onset of therapy to any event (relapse/death/abandonment of treatment against medical advice) with censoring at the time of event or last contact. Continuous variables were represented as mean/median (range) and categorical variables as ratio/proportion. Chi-square test was performed for categorical variables between different clinical, hematological and treatment outcome parameters and genetic events. Survival curves (EFS, RR, OS) for overall cohort in relation to lncRNAs expression were calculated using Kaplan Meier curve and log-rank tests, while data between two groups were evaluated using Mann-Whitney U-test. A p-value of < 0.05 was considered as significant. All statistical analysis was performed using either GraphPad, Prism or SPSS v26.0.

## Results

### Overview of lncRNAs signature in pediatric T-ALL

**Fig. 1:**
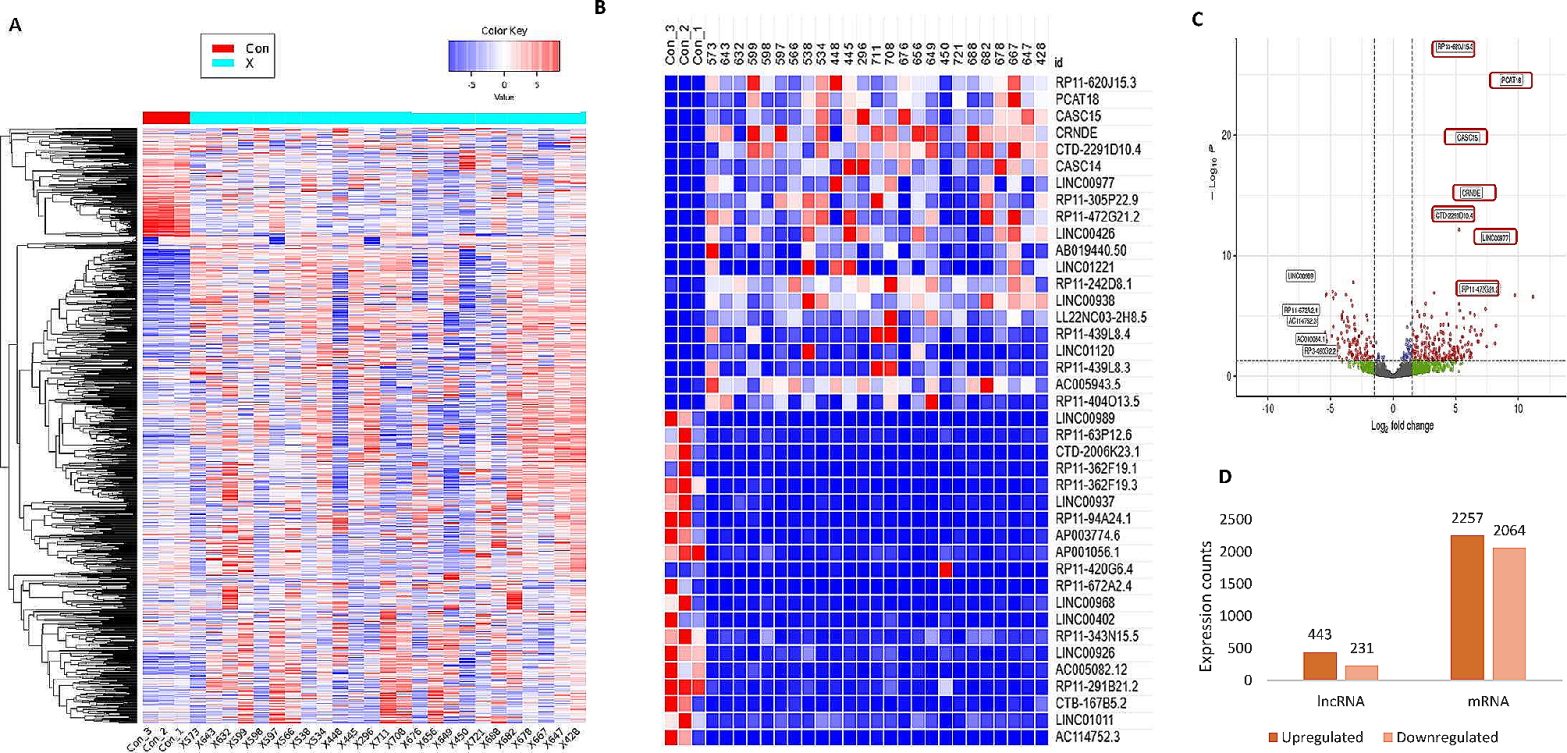
Differential expression of mRNA and lncRNAs in pediatric T-ALL (*N* = 25). **A**, Cluster analysis of mRNA expression in T-ALL samples ( turquoise) and healthy controls (red). **B**, Pathways associated with upregulated and downregulated mRNA in T-ALL. **C**, Heatmap of Top 20 differentially expressed lncRNAs, upregulated and downregulated (*p* < 0.01, fold-change > 2.0). **D**, Volcano plot of upregulated and downregulated lncRNAs. The horizontal dashed line represents a threshold of adjusted p value (< 0.01). The vertical dashed line represents the thresholds of fold change (> 1.5). Red dots represent the statistically significant differentially expressed lncRNAs. Upregulated lncRNAs validated in discovery cohort are outlined in red colour. **E**, Bar plot representing total number of upregulated and downregulated lncRNAs and mRNAs in T-ALL.

To gain an insight into the expression profile of lncRNAs in pediatric T-ALL patients, we performed RNA sequencing analysis in 25 pediatric T-ALL patients (discovery cohort). On differential expression analysis between the patients and controls, we found significant dysregulation of protein-coding genes and lncRNAs signatures (adjusted p value < 0.05, Fold Change > 2) as shown in Fig. 1A (DeSeq2 data is provided in supplemental file 1). Heatmaps and volcano plots are shown in Fig. 1 detailing the distribution of mRNAs and lncRNAs between healthy and T-ALL patients samples. Based on differential gene expression we found 2257 upregulated genes and 2064 downregulated genes respectively, whilst the number of lncRNAs upregulated and downregulated were 443 and 231, respectively (Fig. 1D). Top 12 lncRNAs upregulated in T-ALL (Fig. 1B) were selected for validation in discovery and validation cohort by qRT-PCR. Out of which, seven lncRNAs highlighted in volcano plot (Fig. 1C) showed correlation with RNA sequencing data and were selected for further validation and different analysis.

### Functional prediction of lncRNAs in Cancer pathogenesis

**Fig. 2:**
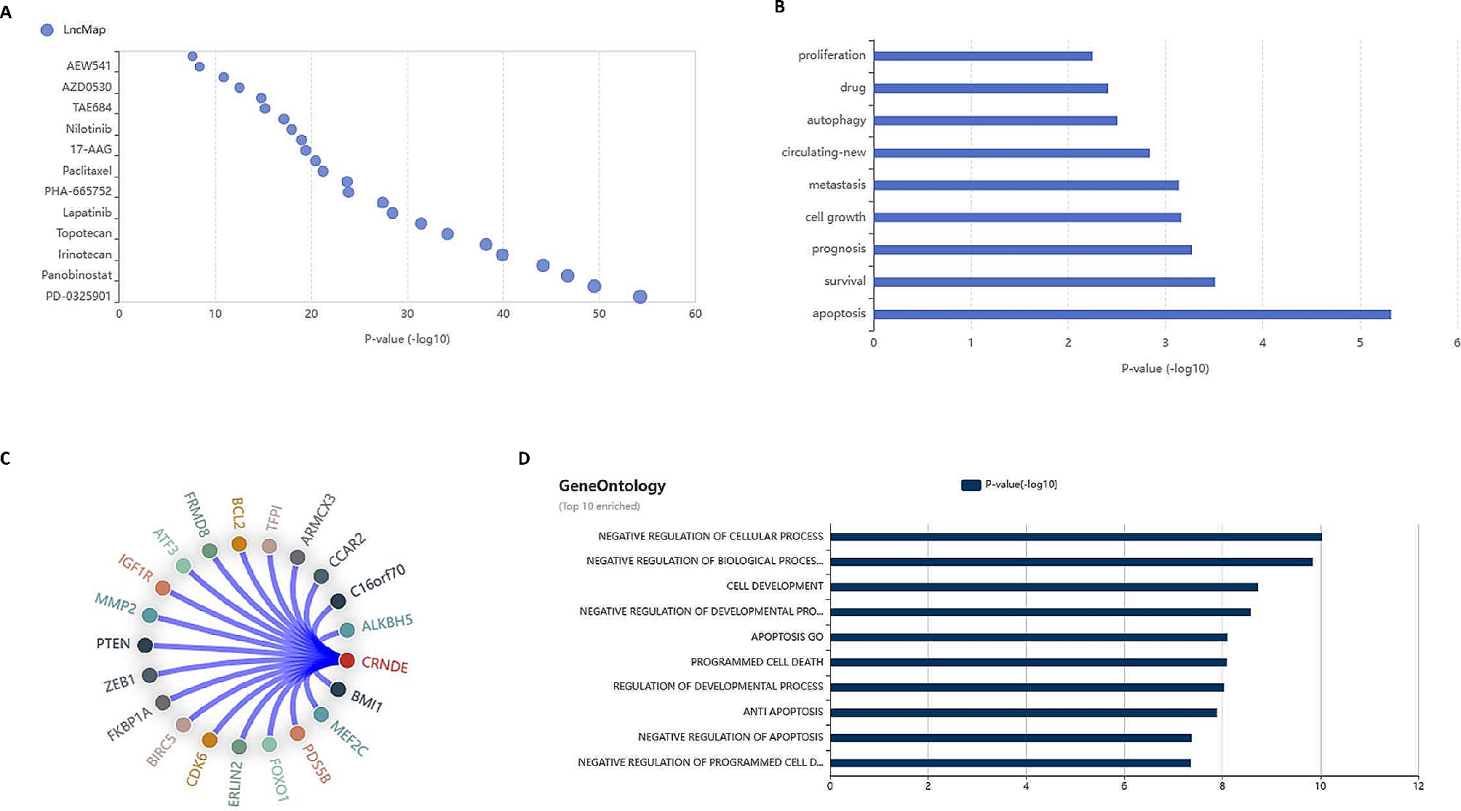
Molecular pathways involved in upregulated lncRNAs. **A**, Relationship between upregulated lncRNAs and drugs curated from LncMAP database (on y-axis, name of drug, x-axis, p values) **B**, List of cancer pathways associated with upregulated lncRNAs. **C**, Interaction map of CRNDE with downstream-located mRNAs calculated by guilt by association strategy using LnCCell database. **D**, Top ten Gene ontology (GO) enriched pathways associated with CRNDE.

We investigated the putative biological functions of the upregulated lncRNAs between pediatric T-ALL and healthy control samples. Due to the lack of functional annotation of lncRNAs, we performed a Gene Ontology (GO) analysis on the protein-coding genes that were predicted to be functionally associated with the differentially expressed lncRNAs. We used database lncSEA to predict the functional associations of these lncRNAs in cancer pathogenesis and drug interactions [27]. This revealed that these lncRNAs interact with various drugs such as paclitaxel, nilotinib, topotecan etc. and may have a significant role in apoptosis, survival and prognosis beside proliferation and metastasis that are characteristics of cancer development and progression (Fig. 2A and B). Out of all the lncRNAs analysed, LINC01221 and CRNDE were the most common to be involved in almost all notable pathways. So, we sought out that whether they have a specific role in T-ALL progression. Using the tool LnCeCell [35], we predicted the pathway enrichment and interaction of these two lncRNAs and found that CRNDE has a significant role in apoptosis, cell development, and programmed cell death (Fig. 2D). It also revealed CRNDE interacts with specific mRNAs dysregulated in cancer progression such as CDK6, PTEN, ALKBH5, IGF1R etc. Among the dysregulated mRNA such as ZEB1, PTEN and MEF2C are known to be specifically involved in T-ALL pathogenesis (Fig. 2C). The database had no data curation for LINC01221, therefore could not be analysed further.

### Interaction of lncRNAs with Coding genes and Biological pathways Analysis

**Fig. 3:**
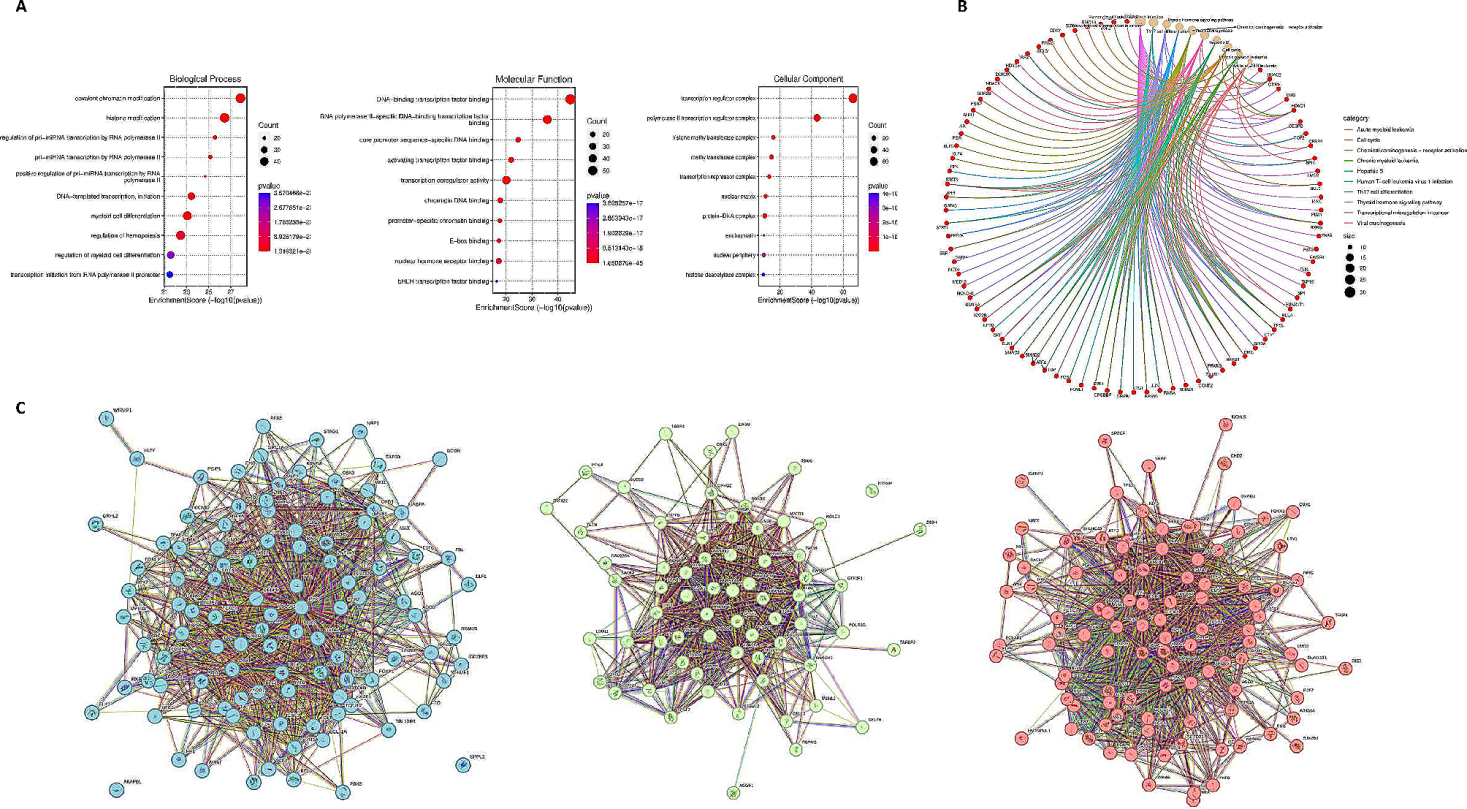
Gene Ontology (GO) analysis of the mRNAs associated with the differentially expressed lncRNAs. **A**, GO analysis of top 10 enriched GO terms for Biological Process (BP), Molecular function (MF) and Cellular Components (CC). **B**, Kegg pathway analysis of the genes associated with upregulated lncRNAs. **C**, STRING protein-protein interaction network showing protein clusters into pathways involved in transcriptional regulation (blue), cell division and differentiation (green) and DNA methylation and demethylation (red). Nodes represent genes in each cluster

Although lncRNAs participate in important functions of cells and play important roles in the pathogenesis of various diseases, but the functional annotation is still lacking. There are many novel lncRNAs, which are dysregulated in cancer, yet data on how they cause the disease lacks. We reasoned that if lncRNAs are crucial for cancer, then their linked genes ought to be over-represented in cancer-related pathways. Hence, we used tools NPinter and ENCORI to look for interaction of these lncRNAs with other protein coding genes. This revealed a total of 288 mRNAs (supplemental file S2). Using these mRNAs, we performed gene ontology and KEGG pathway analysis. In the GO enriched terms in Biological process, molecular function and cellular component, the top enriched pathways were regulation of hematopoises, myeloid cell differentiation, and regulation of transcription. Figure 3A depicts the top 10 results of the GO annotation while supplemental file S3 provides the details of all the enriched significant pathways. We also carried out KEGG pathway analysis and the top five regulated pathways were Human T-cell Leukemia Virus Infection, Thyroid Hormone Signaling Pathway, Th17 cell differentiation, and Chemical Carcinogenesis - Receptor Activation. (Fig. 3B). Further, protein-protein interactions (PPI) network was developed using STRING to cluster proteins into phenotypically relevant modules. The proteins were clustered into three groups using kmeans clustering showing predominantly pathways involved in transcriptional regulation, cell division and differentiation, and DNA methylation and demethylation (Fig. 3C). Nodes in each pathways represent genes shown in different colours. The list of genes clustering together is provided as supplemental file 3. We also performed pathway enrichment analysis using EnrichR and Reactome database (supplemental Figs. 1 and 2). EnrichR curates the data from different databases and the results of six databases Reactome 2022, BioPlanet, NCI-Nature, Elsevier Pathway Collection, Panther 2016 and MiSigDB Hallmark are enlisted in supplemental Fig. 2 [36–38].

### Clinical characteristics of the patients

**Fig. 4:**
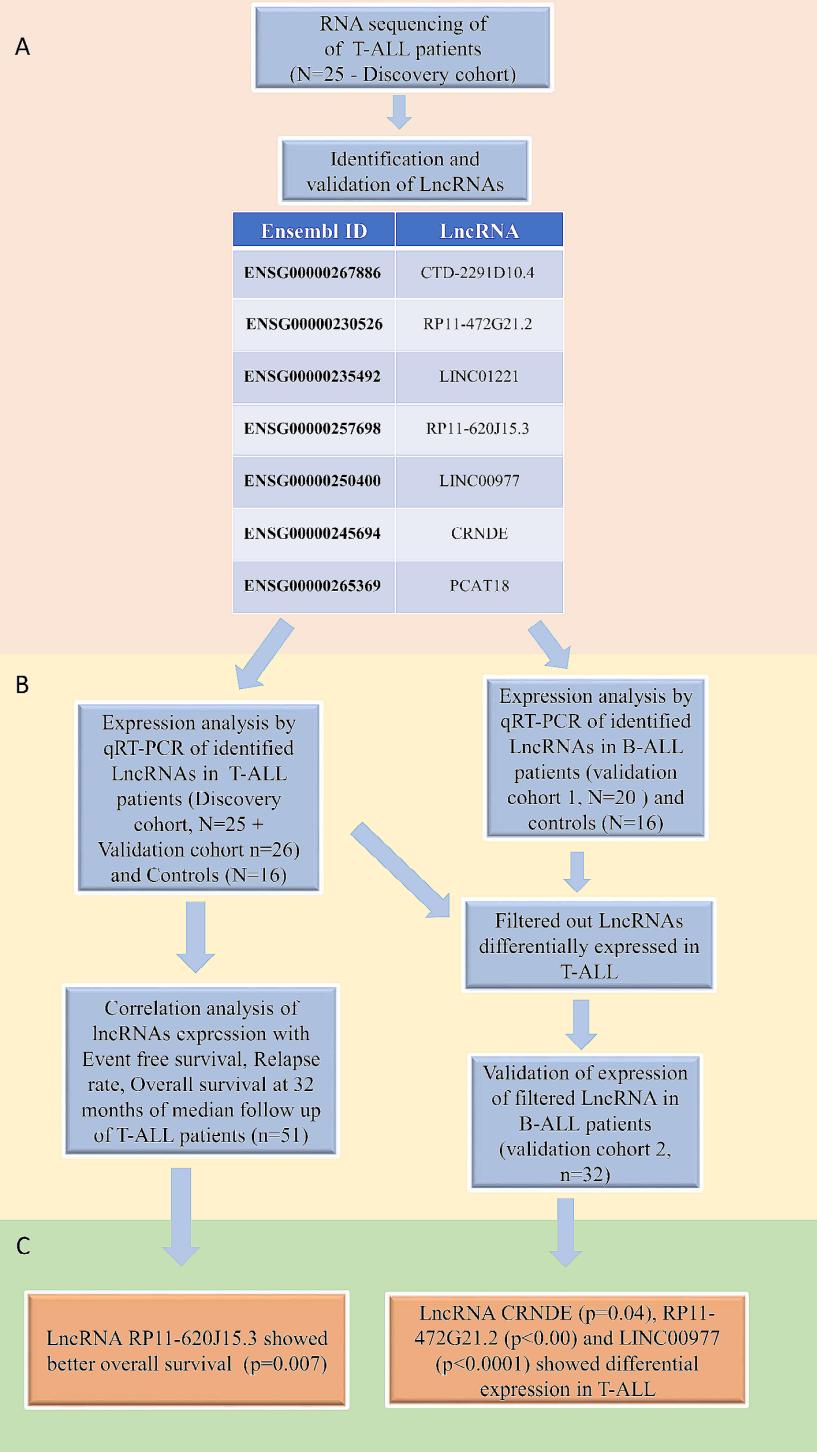
Schematic diagram of identification and validation of lncRNAs. **A**, RNA sequencing analyses in discovery cohort identified list of lncRNAs overexpressed in T-ALL. **B**, Validation of lncRNAs by qRT-PCR in T-ALL and identification of differential expressed lncRNAs in T-ALL vs. B-ALL. **C**, Clinical correlation and survival analysis identified RP11-620J15.3 as predictor of better survival while CRNDE, RP11-472G21.2 and LINC00977 were identified as differentially expressed in T-ALL.

A total of 51 consecutive pediatric cases (25 discover and 26 validation cohort) of T-ALL were enrolled for the study. A schematic diagram of case enrolment for discovery and validation cohort is shown in Fig. 4. The median age of the patients at diagnosis was 7 years (range 3–12 years) with male to female ratio of 12:1. The median WBC count was 168.69 × 10^9^/L (range 27–829 × 10^9^/L). Immunophenotypically, 55% (26/47) cases had a cortical immunophenotype, while 23% (11/47) had mature and 15% (7/47) had pre/pro- T-ALL sub-type, respectively. 6% (3/47) of patients showed early T-precursor (ETP) immunophenotype. Immunophenotype data was unavailable for 4 patients. A good prednisolone response with day 8 absolute blast count (ABC) < 1000/ul was noted in 60% (26/43) cases while 40% (17/43) had a poor prednisolone response. Data for day 8 ABC was not available of 8 cases. The median follow-up duration was 32 months. Nine patients relapsed, eight patients died before completion of therapy. Four patients had lost follow up or left treatment against medical advice. The clinical characteristics of the patients are detailed in Table 1.

**Table 1:** Clinical characteristics of paediatric T-ALL cohort (*N* = 51). ^*^Event = relapse/death/leaving treatment against medical advise

Category (*N* = 51)	T-ALL (n)	T-ALL (%)
Age (range 1–12 years)		
< 5 Years	11	22
> 5 years	40	78
**Gender**		
Male	47	92
Female	4	8
**WBC (range 26.8–829 × 10** ^**9**^ **/L).**		
< 50 × 10^6^/L	4	8
50–100 × 10^6^/L	10	20
> 100 × 10^6^/L	37	72
**Immunophenotype(*N*** **=** **47)**		
Pre-/Pro-TALL	7	15
Cortical/Mature	37	79
ETP	3	6
**Day 8 absolute WBC count(*N*** **=** **43)**		
> 1000 /ul	26	60
< 1000/ul	17	40
**Relapse**		
Yes	9	18
No	42	72
**Death**		
Yes	7	17
No	44	83
**Event** ^*^		
Yes	20	39
No	31	61

### Validation of identified lncRNAs in T-ALL patients

To validate the RNA sequencing result, we enrolled another set of 26 T-ALL patients (validation cohort). We performed qRT-PCR based expression analysis of upregulated lncRNAs in total 51 T-ALL patients (25 from discovery cohort and 26 from validation cohort). Moreover, 16 aged match healthy controls were also enrolled for data normalisation. We performed qRT-PCR validation for top 12 lncRNAs from the list of top 20 upregulated lncRNAs based on adjusted p value < 0.001 and fold change > 2. Validation was performed on the discovery cohort at first (*N* = 25). A set of 7 lncRNAs viz. LINC01221, PCAT18, LINC00977, RP11-620J15.3, RP11-472G21.2, CTD-2291D10.4 and CRNDE were selected that showed correlation with the RNAseq data and were highly expressed in T-ALL cases. These lncRNAs were further analysed for their expression in the validation cohort (*N* = 26). Finally, seven lncRNAs as listed in Table 2 with their Ensemble ID, were analysed and correlated with clinical features of 51 pediatric T-ALL patients.

**Table 2:** List of lncRNAs identified and validated in T-ALL patients and T-ALL cell lines

Ensembl ID	LncRNA	T-ALL (*N* = 51) Median FC	CCRF-CEM* FC	PEER* FC	B-ALL Median FC (N)
**ENSG00000267886**	CTD-2291D10.4	105	855.7	471.5	54.2 (*N* = 20)
**ENSG00000230526**	RP11-472G21.2	105	3848.3	282.0	1.60 (*N* = 52)
**ENSG00000235492**	LINC01221	88	6191.2	3.5	0.51 (*N* = 52)
**ENSG00000257698**	RP11-620J15.3	6.3	27.4	17.9	2.5 (*N* = 20)
**ENSG00000250400**	LINC00977	3.0	33.8	5.3	2.5 (*N* = 20)
**ENSG00000245694**	CRNDE	5.8	7.6	1.5	1.05 (*N* = 52)
**ENSG00000265369**	PCAT18	1.59	4.7	2.5	2.5 (*N* = 20)

LncRNA- long non-coding RNA, T-ALL- T cell acute lymphoblastic leukemia, FC-Fold Change, B-ALL- B-cell acute lymphoblastic leukemia, *T-ALL cell lines

**Fig. 5:**
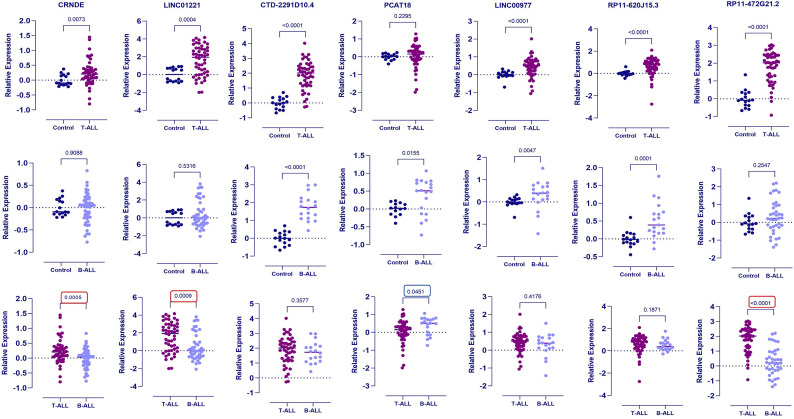
Differential expression of lncRNAs between T-ALL and B-ALL vs. Control validated by qRT-PCR, and the relative expression level of lncRNAs was normalized by U6. **A**, Upregulated lncRNAs as compared to controls. **B**, Upregulated lncRNAs in B-ALL as compared to controls. **C**, Differentially expressed lncRNAs between T-ALL and B-ALL are marked in red (upregulated in T-ALL) and blue (downregulated in T-ALL) colour. The upregulated lncRNAs in T-ALL compared to B-ALL are RP11-472G21.2, CRNDE and LINC01221 while PCAT18 showed higher expression in B-ALL compared to T-ALL.

Based on normalised expression profile of lncRNAs, RP11-472G21.2 and CTD-2291D10.4 were noted to be highly expressed in T-ALL patients (48/51) with a median of fold change of 105 in both (range RP11-472G21.2- 0.012-1060; CTD-2291D10.4- 0.5-10306), followed by LINC01221 (26/51) with median fold change of 88 (range 0.01–14,850) among the patients. RP11-620J15.3, CRNDE, LINC00977 and PCAT18, had median fold change of 6.3, 5.8, 3.0 and 1.6, respectively. Number of patients showing higher expression (> median fold change) compared to control is shown in Table 2; Fig. 5A.

We further confirmed our findings in T-ALL cell lines PEER and CCRM-CRF. Similar to patients’ samples, all 7 above mentioned lncRNAs showed higher expression in both the cell lines except for PCAT18 which did not show higher expression than controls in PEER cell line (Table 2).

### Identification of long non-coding RNA signature distinguishing ALL sub-type

To identify T-ALL specific lncRNAs signature, we further analysed the above 7 lncRNAs in 20 pediatric B-ALL cases. Most of the above lncRNAs showed high expression profile in B-ALL too (Fig. 5B). However, we noticed that higher expression of LINC01221, RP11-472G21.2 and CRNDE were not distributed among all B-ALL cases and were not high as T-ALL patients. Considering this, we further enrolled 32 more B-ALL cases to validate this finding (Table 2). In a total 52 cases of B-ALL, LINC01221 (*p* < 0.0001), CRNDE (*p* = 0.04) and RP11-472G21.2 (*p* < 0.001) showed significantly lower expression pattern than T-ALL suggesting their lineage specific over-expression (Fig. 5C). We also noted that PCAT18 showed significantly higher expression in B-ALL cases than T-ALL (*p* = 0.04). The comparison graphs of lncRNAs expression between T-ALL and B-ALL are shown in Fig. 5C. It is also important to note that LINC01221, CRNDE and RP11-472G21.2 lncRNAs showed higher expression pattern only in T-ALL (*p* < 0.0001) and not in B-ALL as compared to control group making it specific to the T-ALL disease.

### Clinical correlation of lncRNAs with patient characteristics of T-ALL

**Fig. 6:**
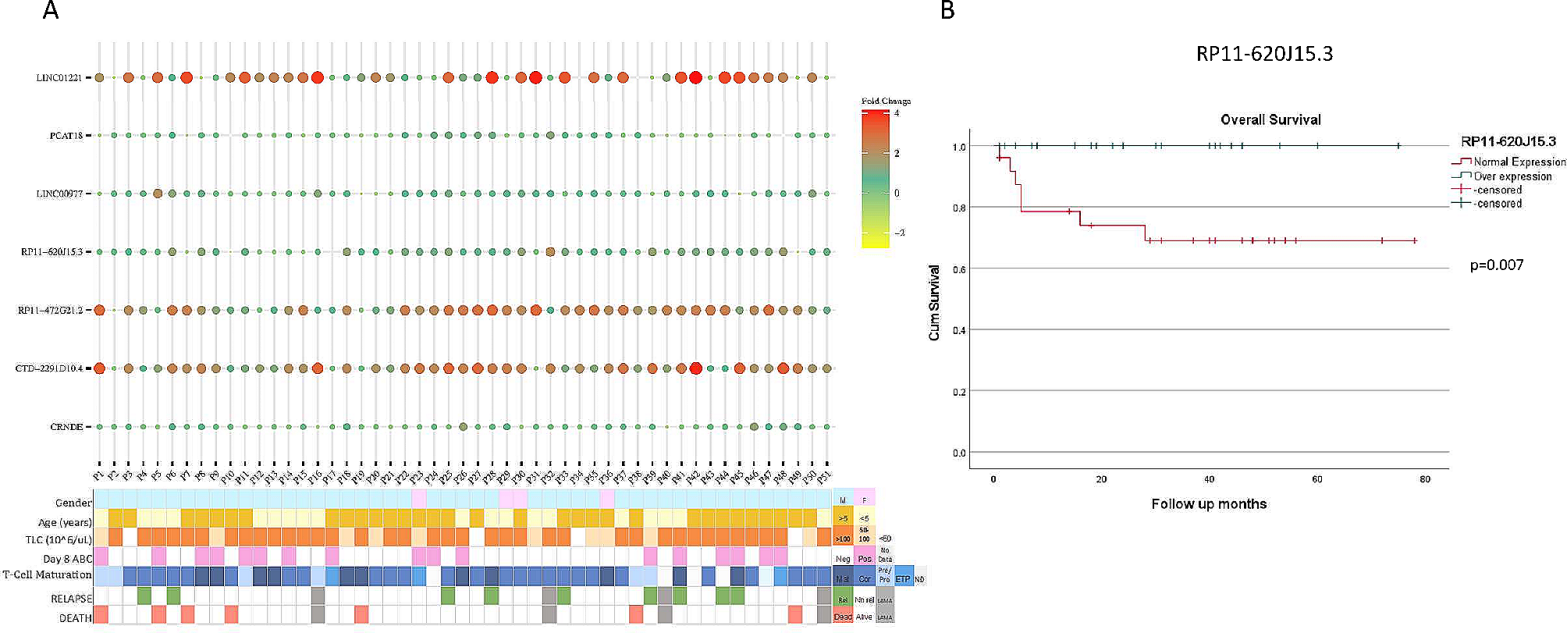
Clinical correlation of lncRNAs with outcomes of pediatric T-ALL patients. **A**, Bubble histogram of identified lncRNAs plotted against clinical characteristics of each patient. TLC, total leukocyte count; ABC, absolute blast count; Neg, Negative; Pos, Positive; Mat, Mature; Cor, Cortical; ND, data not available; Rel, Relapse; LAMA, left treatment against medical advice. **B**, Kaplan Meier survival curve of lncRNA RP11-620J15.3 showing better overall survival at a median follow up of 32 months

To determine the prognostic value of lncRNAs expression, we analysed each lncRNA with pediatric T-ALL clinical data. We tested the correlation of lncRNAs expression with disease prognostic indicators i.e. WBC count at diagnosis, prednisolone response, relapse or death. Expression values in fold change of seven identified lncRNAs is plotted against clinical features and are shown in Fig. 6A. For correlation with WBC count at diagnosis we sub-divided the WBC count into three categories, < 50/51–100/>100 × 10^9^/L. None of the above lncRNAs showed significant correlation with the clinical outcome parameters except RP11-620J15.3 which showed significantly lower cases with deaths (*p* = 0.02) among T-ALL patients (supplementary Table 3).

Further, to determine the prognostic value of lncRNAs, we performed a survival analysis for each lncRNA. To identify differentially expressed lncRNAs in patients with poor outcomes, we considered relapse and death as independent events. Relapse rate (RR) and overall survival (OS) was determined for each lncRNA by Kaplan-Meir curve analysis. Although we did not find any correlation with most of the lncRNAs either for RR or OS (supplementary Figs. 3 and 4), however a very significant association was noted with patients with overexpressed RP11-620J15.3 lncRNA in OS. Patients with over expressed lncRNA RP11-620J15.3 showed better OS for a follow up of 32 months (*p* = 0.007) as shown in Fig. 6B.

## Discussion

Improvements in diagnostics and treatment protocols have improved the outcome for pediatric patients with acute leukemia, with 80% of children undergoing full recovery. However, relapses can lead to poor prognosis, especially in pediatric T-cell leukemias due to their aggressiveness and resistance to standard treatments [39]. Identifying novel targets is crucial for accurate treatment protocols, but childhood T- cell leukemia is heterogeneous. The advent of NGS has boosted the identification of new biomarkers for diagnosis and therapy however, the progress has been uneven. LncRNAs are attracted as potential diagnostic and prognostic biomarkers in leukemia due to their involvement in vital oncogenic processes. This work aimed to bridge this gap and characterize the lncRNA landscape of pediatric T cell acute lymphoblastic leukemia. LncRNAs are a class of biomarkers that modulate gene expression at transcriptional, post-transcriptional, and epigenetic levels. We analysed the expression profiles of lncRNAs using RNA sequencing in pediatric T-ALL and clinically correlated them with patient characteristics.

The expression pattern of lncRNAs in T-ALL patients and healthy controls was examined in this study and we found 674 dysegulated lncRNAs. During the functional enrichment analysis of these lncRNAs, we found that LINC01221 and CRNDE had the highest number of interactions across various pathways associated with cancer progression. While we could not find any other reports about the LINC01221 but the role of CRNDE has been vastly reported in different types of cancer progression. The deletion of the CRNDE locus reduces the proliferative responses to IL6 in multiple myeloma cells [40], and possibly, in a similar manner it may play a role in T-ALL progression, as IL6 signaling is an important part of T-cell differentiation. Though there are no reports on CRNDE expression in T-ALL, a few earlier studies have shown its role in different leukemia. A study by Shehata et al. aimed to check the role of CRNDE in chronic lymphocytic leukemia (CML) and showed that CRNDE is downregulated in CML patients and is associated with poor prognostic markers such as high levels of serum beta-2 microglobulin and lactic dehydrogenase, and the presence of del17p [40]. Expression of CRNDE is also shown to be upregulated in adult acute myeloid leukemia (AML) patients and was associated with lower complete remission and shorter event free survival [41]. Functional studies on KG-1a cell line to explore the role of CRNDE showed that it promotes cell proliferation by modulating the miR-136-5p/MCM5 axis in AML [42]. In another study focussed on expression of CRNDE in AML patients, it was reported [43]. Thus, considering the importance of this lncRNAs in various cancer, our study strengthens its potential to be utilised as biomarker across different malignancies.

Since the roles of the majority of the other differentially expressed lncRNAs were unknown, we undertook GO and KEGG pathway analysis for their functional analysis, which showed that upregulated lncRNAs in T-ALL were associated with the covalent chromatin modification (ontology: biological process), DNA transcription factor binding (ontology: molecular function), transcription regulation complex (ontology: cellular component). According to KEGG pathway analysis, upregulated lncRNAs in T-ALL included Th17 cell differentiation, transcriptional misregulation in cancer and cell cycle pathways. We further validated the RNA sequencing results by analysing the expression of the top upregulated lncRNAs in T-ALL patients. RP11-472G21.2 and CTD-2291D10.4 were highly over expressed in T-ALL patients, followed by LINC01221. All seven lncRNAs under investigation showed higher expression in both T-ALL cell lines, except for PCAT18. The role of PCAT18 in cancer progression remains controversial. PCAT-18 was first discovered to be as a factor involved in invasion, migration, and growth of metastatic prostate cancer [44]. The expression of PCAT18 has been found to vary across different cancer types, highlighting the complexity of the molecular landscape among different tumors. For example, PCAT18 expression is downregulated in gastrointestinal tumor tissues, including gastric cancer [45]. On the other hand, PCAT18 was over expressed more in AML patients with the NPM1 mutation with favorable risk [46]. The expression of PCAT18 in T-ALL has also been reported to be elevated elsewhere, which is in contrast to our findings [47]. Factors contributing to these disparities may include tumor heterogeneity, cellular context, methodological differences, sample size and patient cohorts, stage-specific effects, genetic mutations, and microenvironmental factors. Tumor heterogeneity can influence the expression patterns of PCAT18, while cellular context can influence its function. Single cell-based approach to understand the role of PCAT18 in cancer development and progression may provide more substantial answer to these contradictory results.

We further discovered a distinct lncRNAs signature that could distinguish B-ALL and T-ALL not only from healthy individuals but also from the two forms of leukemia. We chose the potential lncRNA candidates and examined their expression in a broader patient population. The majority of them had their expression absent from the healthy controls and considerable overexpression in a particular kind of ALL suggests a possible diagnostic use in clinical practice. Most lncRNAs showed high expression in B-ALL, while LINC01221, RP11-472G21.2 and CRNDE showed significantly lower expression patterns than T-ALL, suggesting lineage-specific over-expression. A previous study has reported AC247036.1 as differentially expressed lncRNA in T-ALL compared to B-ALL [47]. Further in another study, LINC00152 and LINC01013 are reported to be differentially expressed in B-ALL patients with early relapse and mortality, suggesting lncRNAs can be used as prognostic markers and may also be used as event specific markers [20].

The data analysis of the clinical correlation and prognostic significance of lncRNAs in T-ALL is a key component. RP11-620J15.3 came out as having a substantial connection with overall survival, but the majority of lncRNAs did not demonstrate significant associations with clinical indicators. Patients who expressed this lncRNA more frequently had greater survival rates. This study raises the possibility that RP11-620J15.3 could function as a prognostic marker in children with T-ALL. RP11-620J15.3 has been reported previously in a study by Liu et al. focussed on hepatocellular carcinoma. There in vivo and in vitro studies showed that RP11-620J15.3 play role in cancer proliferation and metastasis by enhancing the Warburg effect through glucose-6-phosphate isomerase in HCC cells [48]. However further detailed studies of pathways in other cancers are still awaited.

The scientific community now has access to a thorough map of the lncRNAs landscape of the various forms of pediatric leukemia, which can be used for therapeutic as well as diagnostic purposes following the proper ad-hoc functional tests. Due to the small sample size, it is nevertheless significant to note the pilot nature of this study. Furthermore, sample size limitations in pediatric research because of logistical and ethical issues, affects statistical power. That could have been one of the reasons of finding scanty prognostic indicators among the over expressed lncRNAs. The other limitation faced were age and gender related biological variability that was not specifically taken into account in our investigation. As in our cohort the male to female ratio was 12:1, that may have produced a biased result on expression pattern or prognostic indication of the identified lncRNAs. However, due to male propensity of the disease and the socio-economy status of the patients taking therapy, these limitations could not be ruled out in our study.

In order to reveal a potential lncRNA’s prognostic role contributing to risk stratification and, consequently, to an improvement in the clinical management of pediatric patients, additional studies with a larger cohort of patients will be required to consistently correlate the expression levels of target lncRNAs to patients’ clinical information. Here, it is significant to emphasize that lncRNAs are simple to find in this regard. Their detection could be included into routine clinical practice, strengthening the diagnostic procedure and enhancing the care of pediatric patients. For example, the differentially expressing lncRNAs in T-ALL patients could be utilised as an additional marker for identifying T-ALL cases with other clinical features to confirm the diagnosis. Further studies can be performed to assess the correlation between the expression of particular candidate lncRNAs such as RP11-620J15.3 for prognostic use in patient stratification that were identified by our investigation and clinical data. Moreover, certain drugs against lncRNAs that are being investigated for their therapeutic role in various cancers could be validated in T-ALL disease too. For example, CRNDE is over expressed in our T-ALL patients and also reported to be increased in other hematopoietic cancers [43]. A study has reported that treatment of human leukemic cell line THP1 with PMA which is a drug to induce terminal monocyte-macrophage differentiation resulted in decreased expression of CRNDE. The expression of CRNDE was also found to significantly downregulated in CML patients after imatinib therapy [49]. These findings show that CRNDE might play an important role in differentiation therapy. Further investigation could be undertaken to explore the efficacy of this drug in T-ALL alone or in combination with other drugs currently being used for T-ALL treatment. Similarly, other significantly over expressing lncRNAs such as RP11-472G21.2, CTD-2291D10.4 and LINC01221 identified in our cohort should be taken up for novel drug designing and validation studies in hematological malignancies. These attributes turned our research into a valuable tool for the scientific community and laid the groundwork for next functional and clinical studies.

## Conclusion

In conclusion, this article presents a comprehensive exploration of lncRNAs in pediatric T-ALL, providing valuable insights into their differential expression, potential functions, and clinical relevance. We identified a distinctive lncRNA signature capable of distinguishing both B-ALL and T-ALL not only from healthy individuals but also among the two sub types of ALL. These differentially over expressed lncRNAs have the potential to be utilised in diagnostic set ups as diagnostic as well as prognostic markers. The identification of T-ALL-specific lncRNAs and the discovery of RP11-620J15.3 as a potential prognostic marker contribute to our understanding of T-ALL and open doors for further research into the role of lncRNAs in leukemia. These findings hold promise for the development of diagnostic tools and therapeutic targets to combat pediatric T-ALL.

### Future directions

The detection of lncRNAs in pediatric T-ALL has unveiled potential biomarkers. Future research should focus on robust functional annotation, longitudinal studies, functional assays, integration with other omics datasets and exploring therapeutic possibilities targeting specific dysregulated lncRNAs. Multi-omic studies, developing biomarker panels, and collaboration with diverse research groups may speed up the research and development in the field of lncRNAs in hematological malignancies which is sparse presently. Embracing these future directions holds the potential to foster the creation of more efficacious diagnostic tools and targeted therapeutic interventions for pediatric T-ALL.

### Electronic supplementary material

Below is the link to the electronic supplementary material.

Supplementary Material 1

Supplementary Material 2

Supplementary Material 3

Supplementary Material 4

Supplementary Material 5

Supplementary Material 6

## Data Availability

All the data from current manuscript has been uploaded as supplementary files.
