# Supplementary material for: Novel lncRNAs LINC01221, RP11-472G21.2 and CRNDE are markers of differential expression in pediatric patients with T cell acute lymphoblastic leukemia
Source: Cancer Cell Int. 2024 Feb 9;24:65. doi: 10.1186/s12935-024-03255-y (PMC10858595; doi:10.1186/s12935-024-03255-y)
Supplement: Supplementary file 1 — Supplementary Material 1 [file 12935_2024_3255_MOESM1_ESM.pptx]

## Slide 1
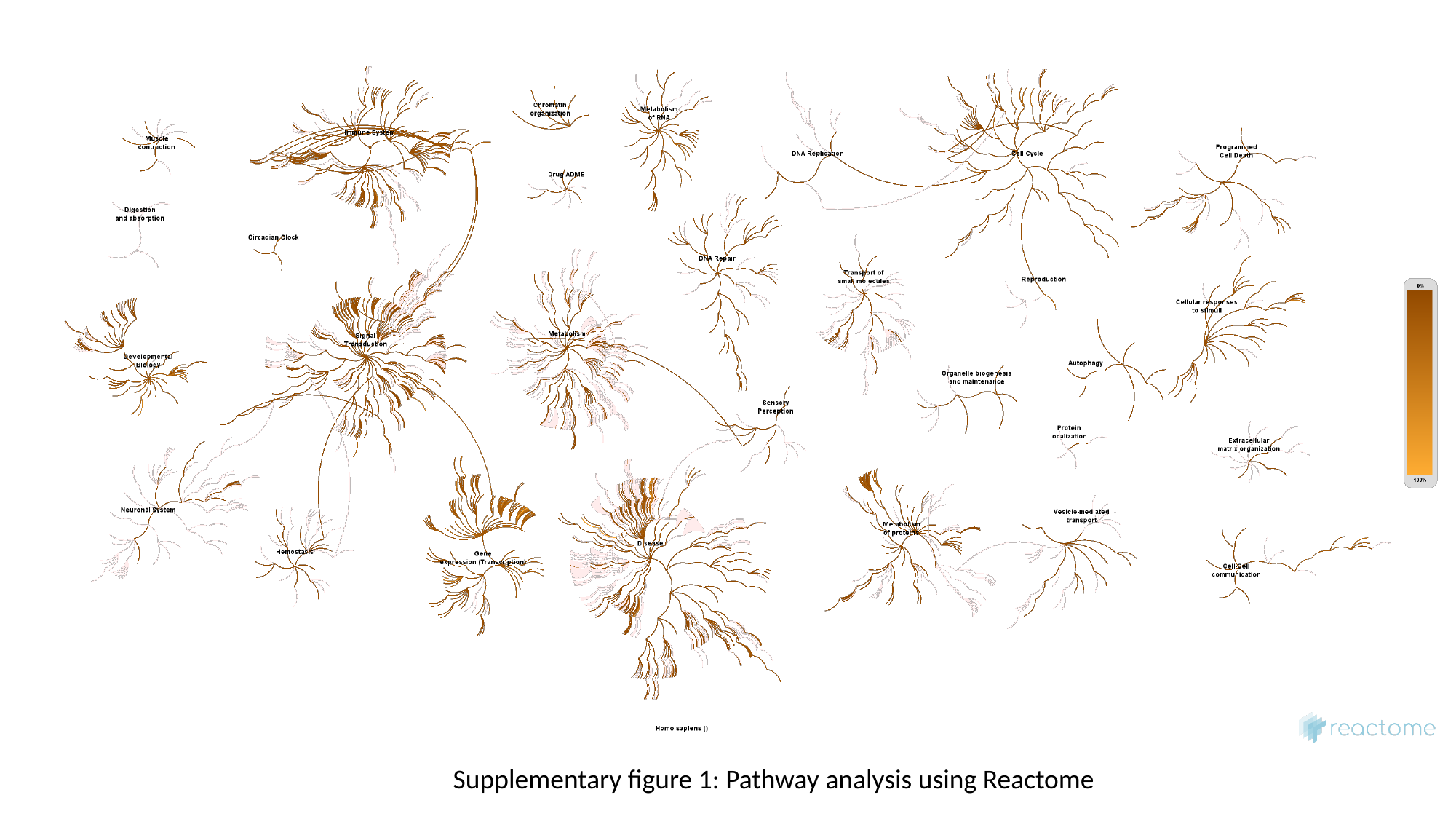

Supplementary figure 1: Pathway analysis using Reactome

## Slide 2
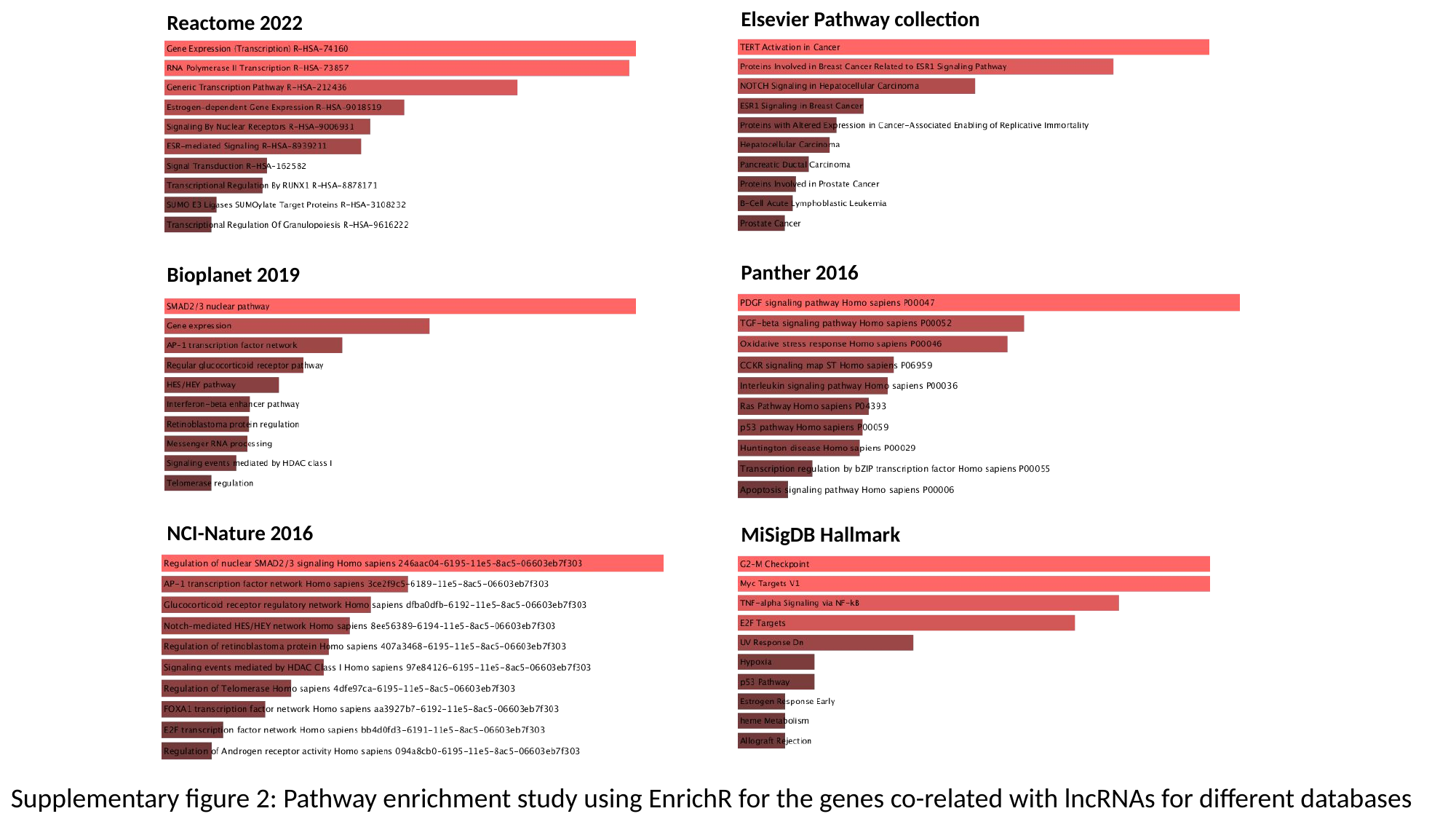

Elsevier Pathway collection
Reactome 2022
Panther 2016
Bioplanet 2019
NCI-Nature 2016
MiSigDB Hallmark
Supplementary figure 2: Pathway enrichment study using EnrichR for the genes co-related with lncRNAs for different databases

## Slide 3
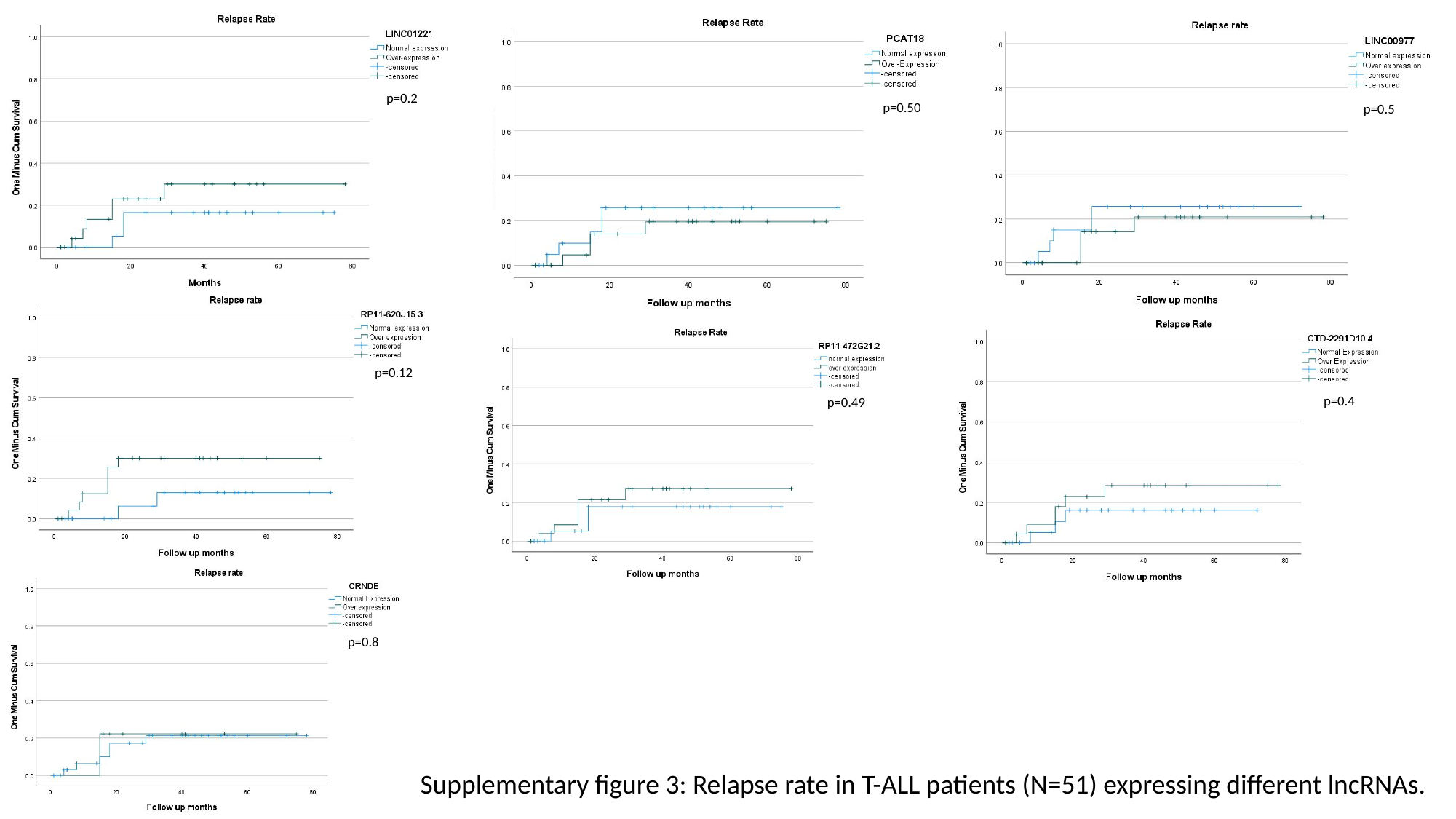

p=0.2
p=0.50
p=0.5
p=0.12
p=0.4
p=0.49
p=0.8
Supplementary figure 3: Relapse rate in T-ALL patients (N=51) expressing different lncRNAs.

## Slide 4
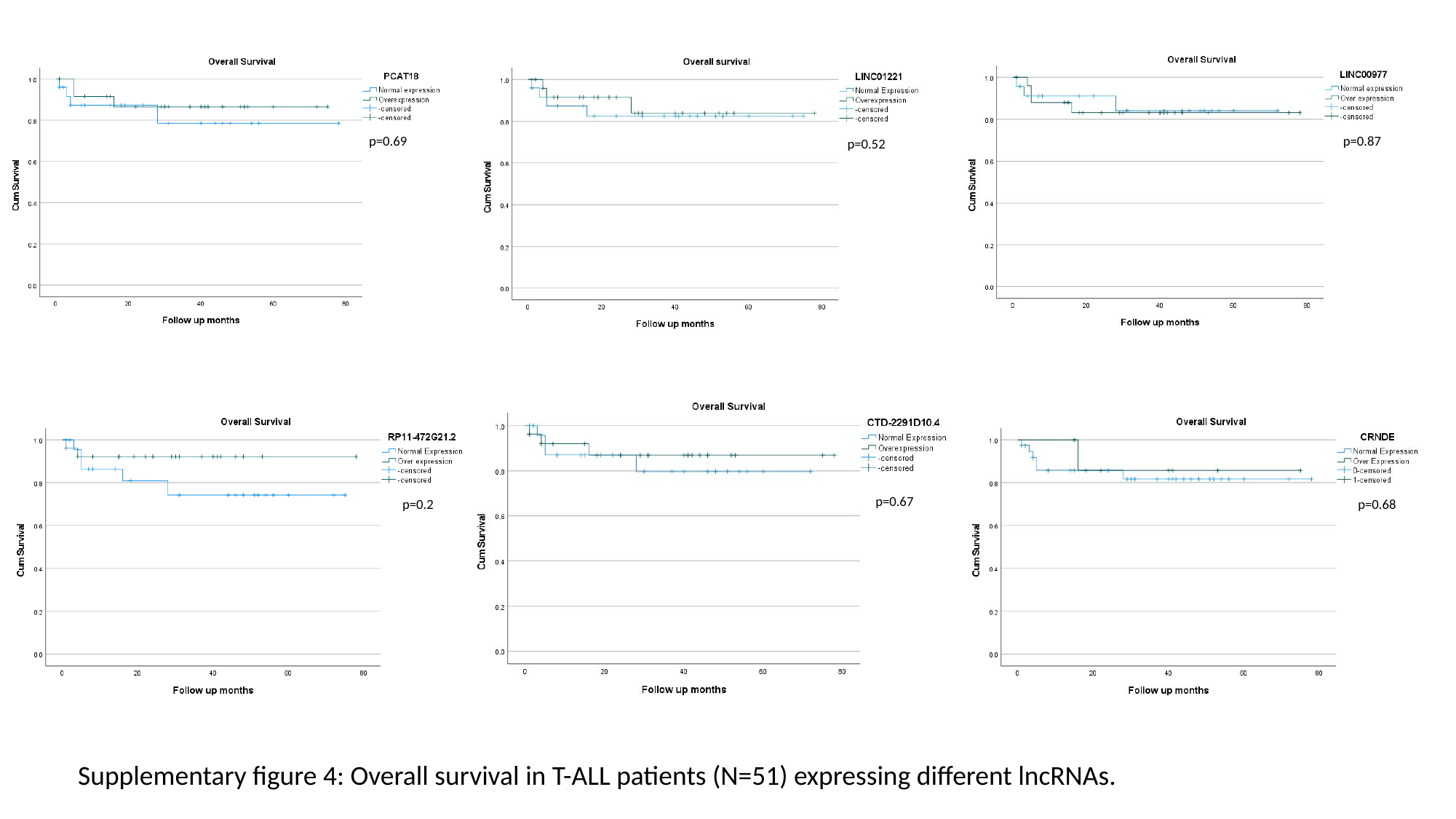

p=0.69
p=0.87
p=0.52
p=0.67
p=0.2
p=0.68
Supplementary figure 4: Overall survival in T-ALL patients (N=51) expressing different lncRNAs.
