# Supplementary material for: Novel lncRNAs LINC01221, RP11-472G21.2 and CRNDE are markers of differential expression in pediatric patients with T cell acute lymphoblastic leukemia
Source: Cancer Cell Int. 2024 Feb 9;24:65. doi: 10.1186/s12935-024-03255-y (PMC10858595; doi:10.1186/s12935-024-03255-y)
Supplement: Supplementary file 2 — Supplementary Material 2 [file 12935_2024_3255_MOESM2_ESM.docx]

| **Name** | Forward | Reverse |
| --- | --- | --- |
| **PCAT18** | TGTTGCTTTCCTGTGACTGC | TCTCAAACTCCCAGGCTCAG |
| **RP11-26E5** | CAGCTGGCCAGAACTACAAA | GTGGGTAAGCAGAAAATGGC |
| **Linc01819** | GAAGAGGCAGCTAGGGAGAG | GTCTTAGGTTCCAGGCCAGT |
| **Linc01120** | CGAGTGTTGCCTCCAAGATG | CTGGAATCCTTCTGCTCCCA |
| **USp13** | AAGCCTCTCATTCACTGGGT | CAGGGGAGCAGGTTTAGACT |
| **CCDC26** | CGTAACCCTGTGCTACCTCA | TGGCAGAAATCCCCAAGTGA |
| **RP11-472G21.2** | CCTCTGGGTTTGTCACAAGC | CGGGGCAGGAAGAAGAACTA |
| **U6** | GCTTCGGCAGCACATATACTAAAAT | CGCTTCACGAATTTGCGTGTCAT |
| **AB019440.50** | TGTTGACTTTTAGGGCGCTG | TGGAGGTCTTCTTAGTGTTCCA |
| **LINC01221** | CCCAAACAGAGGACTCAGCT | CTTGTTCACTGGGCTGTTCA |
| **RP11-236B18.2** | TCCCAGAGAAACGGAGCTTT | ATGGCTTGTTCTCAGGGTCA |
| **AC016735.2** | TGTGGCTGCATCTTCCCATA | CCTTCTGCATCCCTCTTCCA |
| **RP11-620J15.3** | CAAGCGGTTATCCAGTCGTG | AGAGAAGCGACAGTGAGGAG |
| **CASC15** | AGCCAGTTCTAATGCCCACT | CTTCCGATGCCTGCTGTTAC |
| **CTD-2291D10** | ACTCTGTTGCCCTGTGATCT | TCAAATCCCTCTGGTGCTGG |
| **CRNDE** | AAATCAAAGTGCTCGAGTGGTTT | CCTTCTTCTGCGTGACAACTGA |

Supplementary Table 1: List of primers used in validation of lncRNA.
