# Supplementary material for: Novel lncRNAs LINC01221, RP11-472G21.2 and CRNDE are markers of differential expression in pediatric patients with T cell acute lymphoblastic leukemia
Source: Cancer Cell Int. 2024 Feb 9;24:65. doi: 10.1186/s12935-024-03255-y (PMC10858595; doi:10.1186/s12935-024-03255-y)
Supplement: Supplementary file 3 — Supplementary Material 3 [file 12935_2024_3255_MOESM3_ESM.docx]

**Supplementary Table 2: Correlation of lncRNAs with clinico-hematological and treatment outcome in pediatric T-ALL patients (N=51).**

| **Category** | **Total** | **LINC01221** | **p value** | **PCAT18** | **p value** | **LINC00977** | **p value** | **RP11-620J15.3** | **p value** | **RP11-472G21.3** | **p value** | **CTD-2291D10.4** | **p value** | **CRNDE** | **p value** |
| --- | --- | --- | --- | --- | --- | --- | --- | --- | --- | --- | --- | --- | --- | --- | --- |
|  | **(N)** |  |  |  |  |  |  |  |  |  |  |  |  |  |  |
| **Age** | | | | | | | | | | | | | | | |
| **<5 years** | 11 | 6 | 0.78 | 5 | 0.6 | 6 | 0.9 | 6 | 0.78 | 10 | 0.9 | 8 | 0.2 | 2 | 0.13 |
| **≥5 years** | 40 | 20 |  | 21 |  | 22 |  | 20 |  | 16 |  | 18 |  | 17 |  |
| **Gender** | | | | | | | | | | | | | | | |
| **Male** | 47 | 25 | 0.27 | 24 | 0.9 | 25 | 0.4 | 23 | 0.6 | 24 | 0.9 | 22 | 0.15 | 15 | **0.03** |
| **Female** | 4 | 1 |  | 2 |  | 3 |  | 3 |  | 2 |  | 4 |  | 4 |  |
| **WBC at Diagnosis** | | | | | | | | | | | | | | | |
| **≤50 X 10^9^L** | 4 | 1 | 0.35 | 3 | 0.44 | 4 | 0.4 | 1 | 0.5 | 3 | 0.4 | 3 | 0.6 | 4 | 0.1 |
| **50-100 X 10^9^L** | 10 | 4 |  | 6 |  | 5 |  | 5 |  | 4 |  | 5 |  | 3 |  |
| **>100 X 10^9^L** | 37 | 21 |  | 17 |  | 19 |  | 20 |  | 19 |  | 18 |  | 12 |  |
| **Absolute Blast Count at Day 8 (43)** | | | | | | | | | | | | | | | |
| **<1000** | 26 | 13 | 0.85 | 14 | 0.4 | 14 | 0.4 | 10 | 0.09 | 15 | 0.15 | 10 | 0.19 | 12 | 0.13 |
| **>1000** | 17 | 9 |  | 7 |  | 7 |  | 11 |  | 6 |  | 10 |  | 4 |  |
| **Relapse** | | | | | | | | | | | | | | | |
| **Yes** | 9 | 6 | 0.35 | 4 | 0.6 | 4 | 0.4 | 7 | 0.07 | 6 | 0.35 | 6 | 0.9 | 4 | 0.6 |
| **No** | 42 | 20 |  | 22 |  | 24 |  | 19 |  | 20 |  | 22 |  | 15 |  |
| **Death** | | | | | | | | | | | | | | | |
| **Yes** | 7 | 3 | 0.6 | 3 | 0.6 | 4 | 0.4 | 0 | **0.02** | 2 | 0.2 | 3 | 0.6 | 3 | 0.7 |
| **No** | 44 | 23 |  | 23 |  | 24 |  | 26 |  | 24 |  | 23 |  | 16 |  |
| **Event** | | | | | | | | | | | | | | | |
| **Yes** | 20 | 11 | 0.6 | 9 | 0.6 | 10 | 0.5 | 10 | 0.9 | 8 | 0.2 | 11 | 0.6 | 7 | 0.7 |
| **No** | 31 | 15 |  | 17 |  | 18 |  | 16 |  | 18 |  | 15 |  | 12 |  |
